# Natural killer cell-related prognosis signature predicts immune response in colon cancer patients

**DOI:** 10.3389/fphar.2023.1253169

**Published:** 2023-11-13

**Authors:** Meiqin Li, Jingqing Song, Lin Wang, Qi Wang, Qinghua Huang, Dan Mo

**Affiliations:** ^1^ Department of Clinical Laboratory, Guang Xi Medical University Cancer Hospital, Nanning, China; ^2^ Department of Gastrointestinal Surgery, Guang Xi Medical University Cancer Hospital, Nanning, China; ^3^ Department of Clinical Laboratory, The Fifth Affiliated Hospital of Guangxi Medical University, Nanning, China; ^4^ School of Basic Medical Sciences, Guangxi Medical University, Nanning, China; ^5^ Department of Basic Medicine, Guangxi Health Science College, Nanning, China; ^6^ Department of Breast Surgery, Wuzhou Red Cross Hospital, Wuzhou, China; ^7^ Department of Breast, Maternal and Child Healthcare Hospital of Guangxi Zhuang Autonomous Region, Nanning, China

**Keywords:** TCGA, natural killer cells, GEO, prognosis, colorectal cancer, immunotherapy

## Abstract

**Background:** Natural killer (NK) cells are crucial components of the innate immune system that fight tumors and viral infections. Patients with colorectal cancer (CRC) have a poor prognosis, and immunotherapeutic tools play a key role in the treatment of CRC.

**Methods:** Public data on CRC patients was collected from the TCGA and the GEO databases. Tissue data of CRC patients were collected from Guangxi Medical University Affiliated Cancer Hospital. An NK-related prognostic model was developed by the least absolute shrinkage and selection operator (LASSO) and Cox regression method. Validation data were collected from different clinical subgroups and an external independent validation cohort to verify the model’s accuracy. In addition, multiple external independent immunotherapy datasets were collected to further examine the value of NK-related risk scores (NKRS) in the prediction of immunotherapy response. Potential biological functions of key genes were examined by methods of cell proliferation, apoptosis and Western blotting.

**Results:** A novel prognostic model for CRC patients based on NK-related genes was developed and NKRS was generated. There was a significantly poorer prognosis among the high-NKRS group. Based on immune response prediction, patients with low NKRS may be more suitable for immunotherapy and they are more sensitive to immunotherapy. The proliferation rate of CRC cells was significantly reduced and apoptosis of CRC cells was increased after SLC2A3 was knocked down. SLC2A3 was also found to be associated with the TGF-β signaling pathway.

**Conclusion:** NKRS has potential applications for predicting prognostic status and response to immunotherapy in CRC patients. SLC2A3 has potential as a therapeutic target for CRC.

## Introduction

Globally, colorectal cancer ranks fourth in terms of cancer-related deaths (Gausman et al., 2020; Shaukat et al., 2021; Xi and Xu, 2021). The prognosis of some patients with CRC remains poor despite surgical and medical strategy improvements over the past decade. Cancer cells grow due to intrinsic and extrinsic factors in the tumor microenvironment. Cells from the immune system play a crucial role in the progression and prognosis of CRC due to their position in the TME. In the innate immune system, natural killer (NK) cells are one of the major types of cells that infiltrate tumors and increase the likelihood of a better prognosis in CRC (Becker et al., 2016; Wu et al., 2020a; Guillerey, 2020). NK cells are valuable tools in cancer immunotherapy because of their ability to kill tumor cells in various ways without prior sensitization. The immunological and molecular mechanisms underlying CRC remain largely unclear, which limits our ability to improve its treatment. For the development of targeted CRC therapies, it is, therefore, necessary to identify novel biomarkers. There is evidence of the potential of NK cells to treat CRC, especially when they are engineered with chimeric antigen receptors (CAR) for cancer treatment (Wang et al., 2020; Xie et al., 2020; Wrona et al., 2021). However, the development of NK cell clinical therapies still faces many challenges. Studying NK cell-related genes may help us better understand the landscape of TME in CRC patients.

**FIGURE 1:**
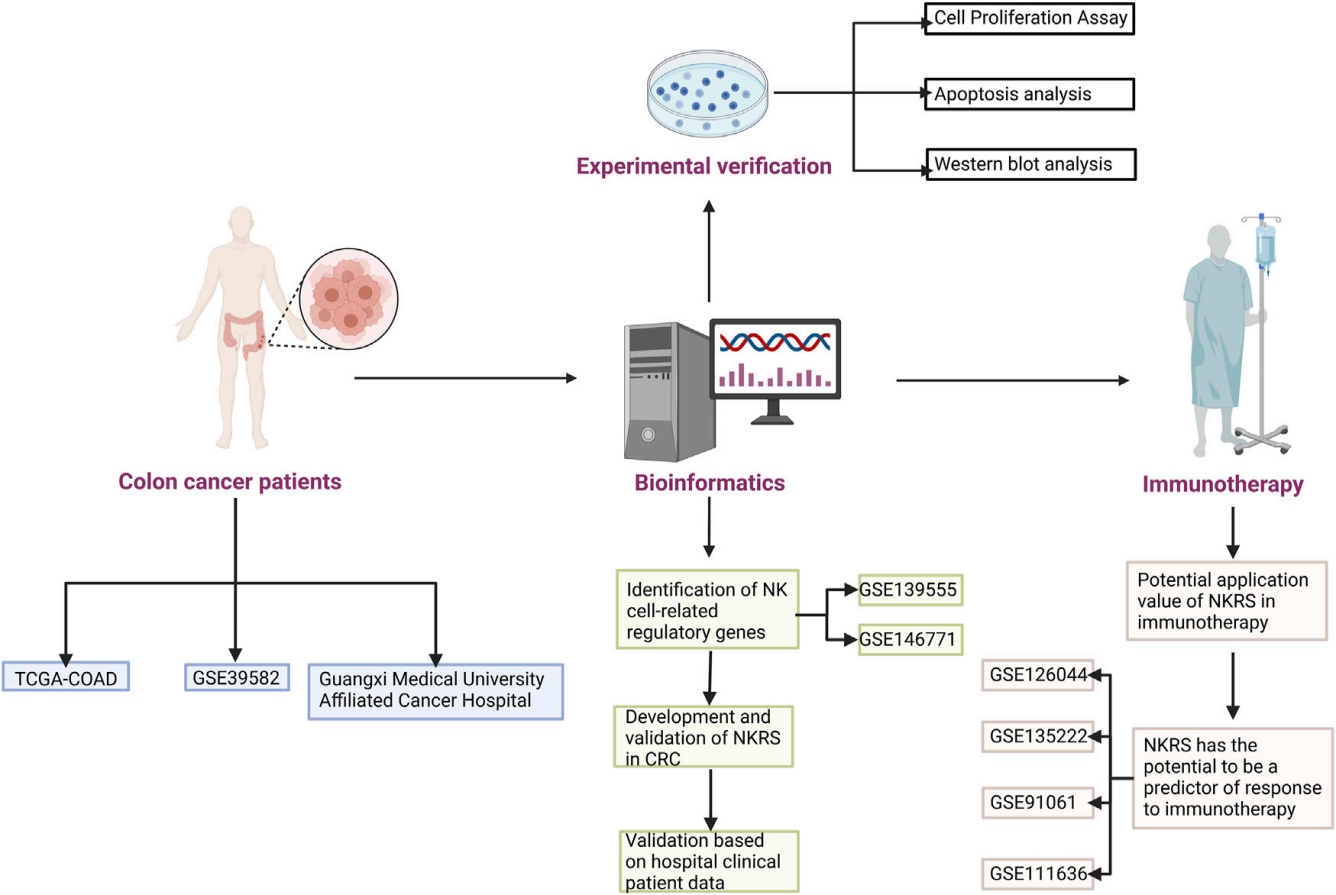
Flow Chart.

Single-cell sequencing has become one of the necessary tools for tumor research. With the results of single-cell sequencing, detailed information on cell subsets and key regulatory mechanisms in tumor cells could be obtained (Mei et al., 2021; Zheng et al., 2021). In this research, the essential regulatory genes of NK cells in CRC patients was mined based on the results of single-cell sequencing. Based on these genes, we constructed a prognostic model for CRC patients and generated NK-related risk scores. Based on the fact that NKRS can differentiate patients, we investigated the differences between high and low NKRS subgroups, and these findings can inform personalized clinical treatment of CRC patients, especially in immunotherapy. In addition, we validated our results in the hospital clinical sample and in multiple independent datasets. Figure 1 shows how the study was designed.

## Methods

### Public data download and processing

Data from the TCGA database were used to analyze CRC patients’ transcriptome data and clinical information (TCGA-COAD) (https://portal.gdc.cancer.gov/repository). Our data were obtained from the TCGA database and included RNA-seq (level 3, HTSeq-FPKM data) for 445 colon cancer patients (41 solid normal tissues and 445 primary tumor tissues) (Ahluwalia et al., 2019; Zhang et al., 2021a; Ma et al., 2022). Samples without clinical information or with a follow-up time of fewer than 30 days were excluded to reduce the interference of irrelevant factors. The GEO database (http://www.ncbi.nlm.nih.gov/geo/) was used to obtain single-cell sequencing data for CRC patients (GSE146771, GSE139555) (Marisa et al., 2013; Zhang et al., 2020). The GSE139555 study examines single-cell TCR-seq and single-cell RNA-seq data from pretreatment samples taken from tumors, adjacent tissues, and peripheral blood of 14 cancer patients. GSE146771, For Patient P1228, P0305, P0728, P0411, P0825, P0104, P0309, P0720, P1212, and P0413, single-cell RNA-seq were performed by the SMART-seq2 platform; For Patient P0123, P0305, P0323, P0410, P1025 P0104, P0202, P0408, P0613, P1026, single-cell RNA-seq were performed by the 10x Genomic single cell 3’ library platform. In addition, external independent immunotherapy datasets (GSE126044, GSE135222, GSE91061, and GSE111636) for multiple cancers were used as a further test of NKRS.

### Validation cohort from hospital

The process for obtaining a validation cohort is as follows: We obtained fresh colon cancer tissues and normal tissues (10–15 cm from the cancer tissues) from 18 colon cancer patients without chemoradiotherapy from Guangxi Medical University Affiliated Cancer Hospital for transcriptome sequencing. All methods were performed in accordance with the relevant guidelines and regulations, and were approved by Guangxi Medical University Affiliated Cancer Hospital Ethical Review Committee (LW2023077). The patients provided written informed consent to participate in this study.

According to the manufacturer’s instructions, the trizol (Ambion, Invitrogen, United States) reagent was used to extract RAN from tissues. The RNA Nano 6000 Assay Kit of the Bioanalyzer 2100 system (Agilent Technologies, United States) was used to measure the value of the RNA integrity number. The library was then established using the NEBNext Ultra RNA Library Prep Kit for Illumina Kit (NEB, United States). The AMPure XP system (Beckman Coulter, United States) was then used to screen fragments, and ABI2720 (Applied Biosystems, United States) was used to build libraries to amplify cDNA. The touch q-PCR system CFX96 (BIO-RAD, United States) was used to detect library concentrations. Finally, the novaseq 6000 (illumina, United States) was used for sequencing.

### Cell culture and siRNA transfection

Colon cancer cells (SW480 and RKO) were purchased from cell bank (Shanghai Institute of Biochemistry and Cell Biology, china) and cultured in 10% FBS+DMEM. From RiboBio Guangzhou (China), siRNA for SLC2A3 was purchased, along with a negative control NC. It follows the following sequence if-SLC2A3: GTA​GCT​AAG​TCG​GTT​GAA​A. A Lipofectamine3000 reagent (Thermo Fisher Scientific) was used for siRNA transfection following the manufacturer’s instructions.

### Cell proliferation assay

Cell viability was determined using CCK-8 (Cell Counting Kit-8, Dojindo Molecular Technologies, Kumamoto), according to the manufacturer’s instructions. Briefly, approximately 2 × 103 cells in the logarithmic growth phase were collected and then seeded into 96-well plates. Then, 10 μL CCK8 reagent was added to each well and incubated at 37°C for 1 h. The absorbance values at 450 nm were measured at 24, 48, and 72 h using the Benchmark microplate reader (BioTek Instrument, Inc., Winooski, VT, United States).

### Western blot

In this study, we used RIPA buffer from Beyotime (Shanghai, China) to extract proteins from cells. After 48 h of siRNA transfection, cells were collected. The antibodies include, TGF β1 (1:2000), Smad2 (1:2000), SLC2A3 (1:1000), TGF β2 (1:1000)), Smad3 (1:1500), phospho-Smad2 (1:1000 and phospho-Smad3 (1:2000). Antibodies used in this study were purchased from Abcam Inc. (Cambridge, MA, United States).

### Identifying NK cells related differential expressed genes

A database of single cells derived from tumors is called the Tumor Immune Single-cell Hub (TISCH) (Sun et al., 2021). Using TISCH as a database, we extracted NK Cells-related genes from the single-cell data of CRC patients (Ning et al., 2022; Zhang et al., 2022). The limma package was used to screen COAD and normal colon samples for mRNA expression. The criteria were |log 2(fold change) |>1 and a false discovery rate (FDR) < 0.05.

### Establishment of the risk model

By using Univariate Cox regression, NKGs significantly different from each other were analyzed for their association with OS in CRC patients. LASSO regression analysis was performed on these NKGs to identify more significant prognostic factors. Based on two NKGs significantly associated with OS, patients were divided into high-risk and low-risk subgroups using the median risk score. ROC curves were used to test the model’s accuracy. Moreover, the GSE39582 validation cohort was used to validate the model.

### Independent prognostic analysis and principal component analysis

In our study, we examined whether clinical characteristics were independent prognostic factors. A multivariate independent prognostic and Cox regression-based univariate study was conducted using the “survival” package (Ding et al., 2021; Yue et al., 2021). By employing NKG expression patterns and principal component analysis, the dimensionality of the high-dimensional gene expression profile was weakened, the model identified, and the data visualized.

### Functional analysis

We used the “GSVA” R package to analyze GSVA enrichment data to compare high-risk and low-risk biological processes. We used R package cluster profiles for functional annotation and MSigDB gene set (c2.cp.kegg.v7.2.symbols.gmt) for gene symbol annotation (https://www.gsea-msigdb.org) (Subramanian et al., 2005; Chen et al., 2019; Ji et al., 2020). Comparing low- and high-RS subgroups were quantified by differential immune cell infiltration and functional immunological enrichment using the ssGSEA algorithm.

### Analysis based on online websites

UALCAN is an OMICS data analysis web service (http://ualcan.path.uab.edu/index.html) that provides comprehensive OMICS data analysis for cancer (Chandrashekar et al., 2022). A UALCAN analysis was performed on key genes to determine their protein expression levels. TIMER (https://cistrome.shinyapps.io/timer/) is a fast web application that analyzes immune infiltration in various cancer types worldwide (Li et al., 2017). Based on the TIMER algorithm, gene expression was correlated with immune infiltration. The TIMER algorithm was used to predict the correlation between gene expression and immune infiltration.

### Analysis of the potential value of NKRS in immunotherapy

The Cancer Immunome Database (TCIA) provides results of comprehensive immunogenomic analyses of next-generation sequencing data (NGS) data for 20 solid cancers from TCGA and other datasources (Charoentong et al., 2017). We calculated Immuno appearance scores (IPSs) based on data from CRC patients in TCIA to predict the response of patients to immunotherapy. Then, we employed the Tumor Immune Dysfunction and Exclusion (TIDE; http://tide.dfci.harvard.edu/) to predict the immune response of high- and low-NKRS subtypes (Fu et al., 2020). In addition, we obtained immunotherapy datasets from the GEO database for different types of cancer and the predictive value of NKRS was further validated.

### Statistical analysis

Data was analyzed using R version 4.1.2. Analyses were performed using the Student’s t-test (two-tailed, unpaired) to compare the two independent groups (Wu et al., 2021; Guo et al., 2022; Lu et al., 2022; Périchon et al., 2022). Clinical characteristics were compared according to their proportions using the chi-squared test. When more than two groups were present, one-way analysis of variance and Kruskal–Wallis tests were used as non-parametric and parametric methods. R software GSVA package was used to analyze, choosing parameter as method = “ssgsea”. The correlation between genes and pathway scores was analyzed by Spearman correlation. *p* < 0.05 was deemed to be significant.

## Result

### Identification of NK cell-related regulatory genes in CRC based on single-cell data

**FIGURE 2:**
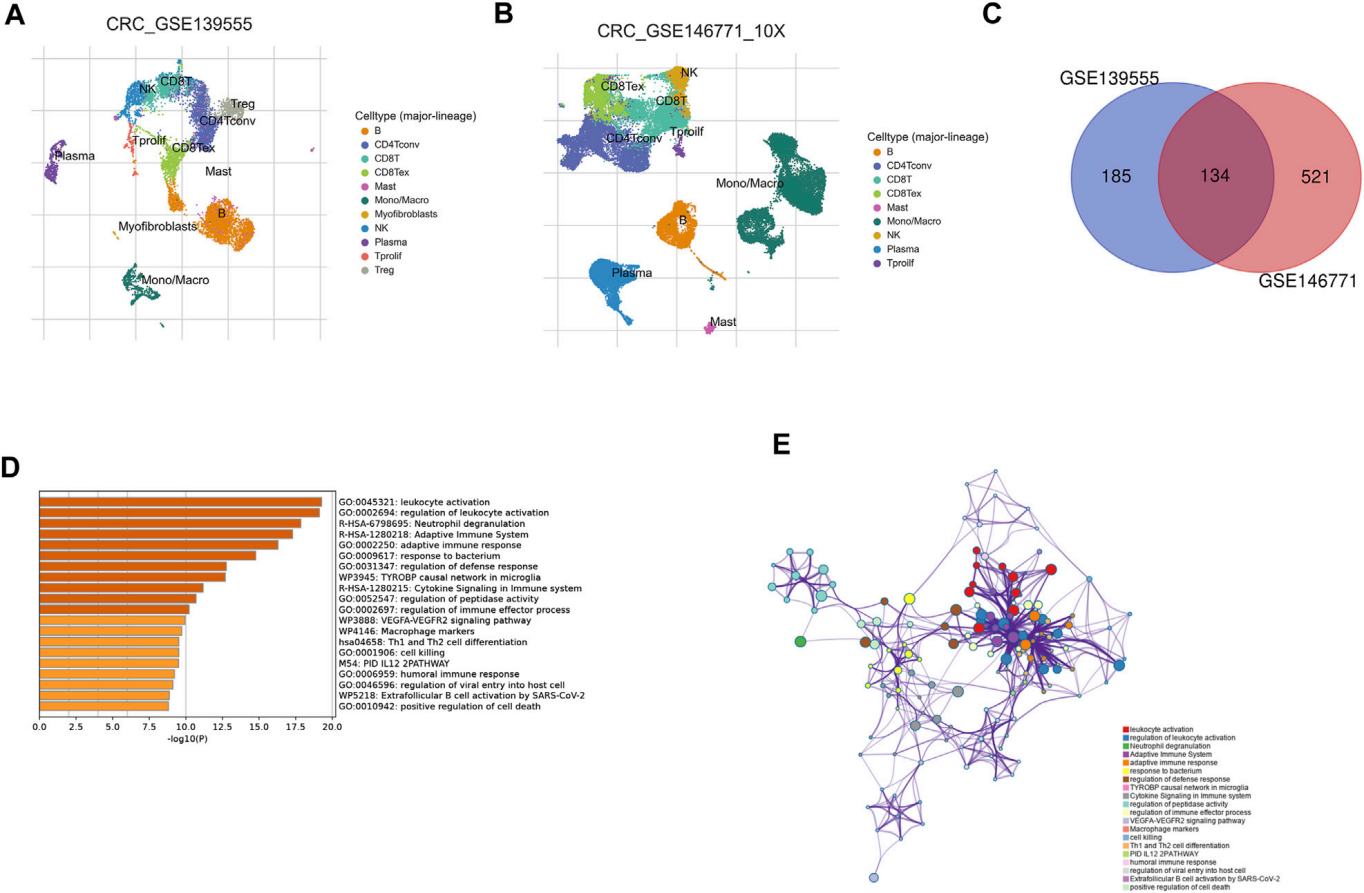
Identification of NK cell-related regulatory genes in CRC based on single-cell data. **(A)** UMAP plot showing cell type coloring in GSE139555 data. **(B)** UMAP plot showing cell type coloring in GSE139555 data. **(C)** Venn diagram showing key NKGs. **(D)** Metascape enrichment analysis for the NKGs. **(E)** Metascape enrichment network visualization showing the intra-cluster and inter-cluster similarities of enriched terms.

Single-cell analysis has been widely used in the study of various cancers. Single-cell analysis of GEO data enabled us to visualize the significant subsets of CRC cells (Figure 2A, GSE139555; Figure 2B, GSE146771) and identified NK cell-related regulatory genes (NKGs). The NKGs determined by the two single-cell data were further merged, and the overlapping genes were regarded as NKGs that better fit our requirements (Figure 2C; Supplementary Table S1). As a result of the functional enrichment analysis conducted by Metascape, these NKGs are mainly enriched in functional pathways related to such as humoral immune response, immune regulation, regulation of leukocyte activation, leukocyte activation, adaptive immune response, Cytokine Signaling in the Immune system, and VEGFA-VEGFR2 signaling pathway (Figure 2D). The regulatory relationship between the pathways is shown in Figure 2E.

### Development and validation of NKGs prognostic risk signatures in CRC

**FIGURE 3:**
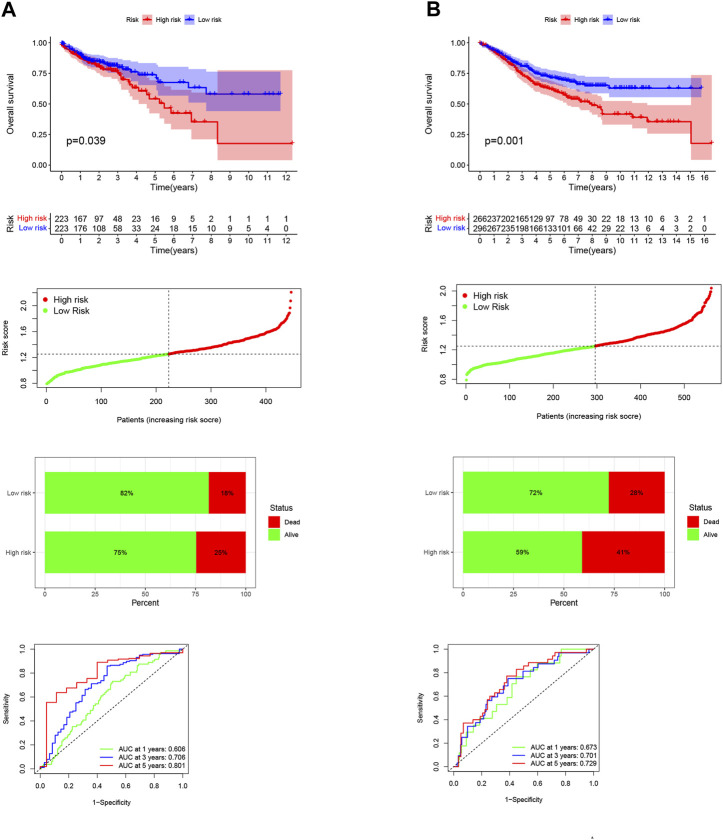
Development and validation of NKGs prognostic risk signatures in CRC. **(A)** Training cohort. **(B)** Validation cohort. From top to bottom: Kaplan-Meier survival analysis of high- and low-RS subgroups; risk score between high- and low-risk groups; survival status of patients in high- and low-RS subgroups; plots of the AUC for time-dependent ROC performance.

**FIGURE 4:**
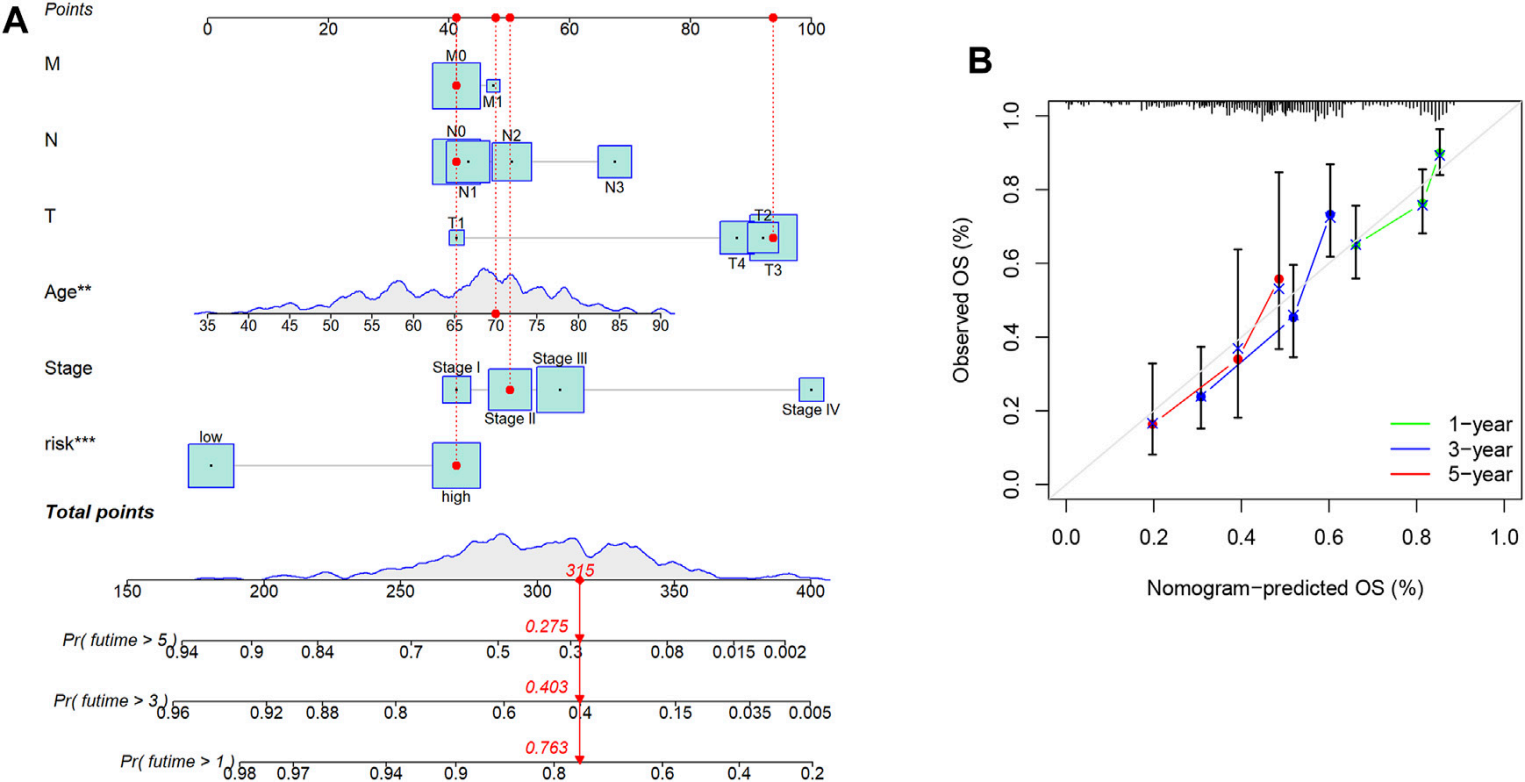
Nomogram Analysis. **(A)** The nomogram to predict the 1-year, 3-year, and 5-year overall survival rate of colon cancer patients. **(B)** The calibration curve for evaluating the accuracy of the nomogram model. The dashed diagonal line in grey colour represents the ideal nomogram.

Differential gene analysis was first performed in the TCGA-COAD dataset and further screened for significantly different NKGs (Supplementary Figures S1A, B). A prognostic model was then constructed using LASSO (*SLC2A3*+*POU2F2*) of CRC and generating an NK-risk score (NKRS) (Supplementary Figures S1C, D). High- and low-risk subgroups were divided using median RS values. The results of PCA analysis showed that RS could better classify patients into two different subtypes (Supplementary Figure S2). Moreover, we found that patients in the high-RS group were associated with poorer prognosis and had a higher rate (25%) of death (Figure 3A). The NKGs-related model had high accuracy in CRC patients. Based on ROC curves that are influenced by time, the model’s predictive ability in CRC patients was assessed. The AUCs at 1, 3, and 5 years were 0.606, 0.706, and 0.801, respectively. To further validate the accuracy of the NKGs model, the mRNA expression matrix (GSE39582) was downloaded from another CRC cohort in the GEO database for use as a validation cohort. TCGA-COAD findings confirm that patients in the high-RS subgroup have worse outcomes and have a higher death rate than patients in the low-RS subgroup (Figure 3B). In addition, the AUCs of the GSE39582 cohort at 1, 3, and 5 years was 0.673, 0.701, and 0.729, respectively. AUC results further validate the model’s accuracy. In addition, we used a nomogram to sum the scores of multiple correlated factors to predict further the likelihood of survival in CRC patients (Figures 4A, B). Based on the nomogram results, our RS was also shown to be valuable in CRC patients.

### Expression levels of NK cell-related regulatory genes and NK correlation

**FIGURE 5:**
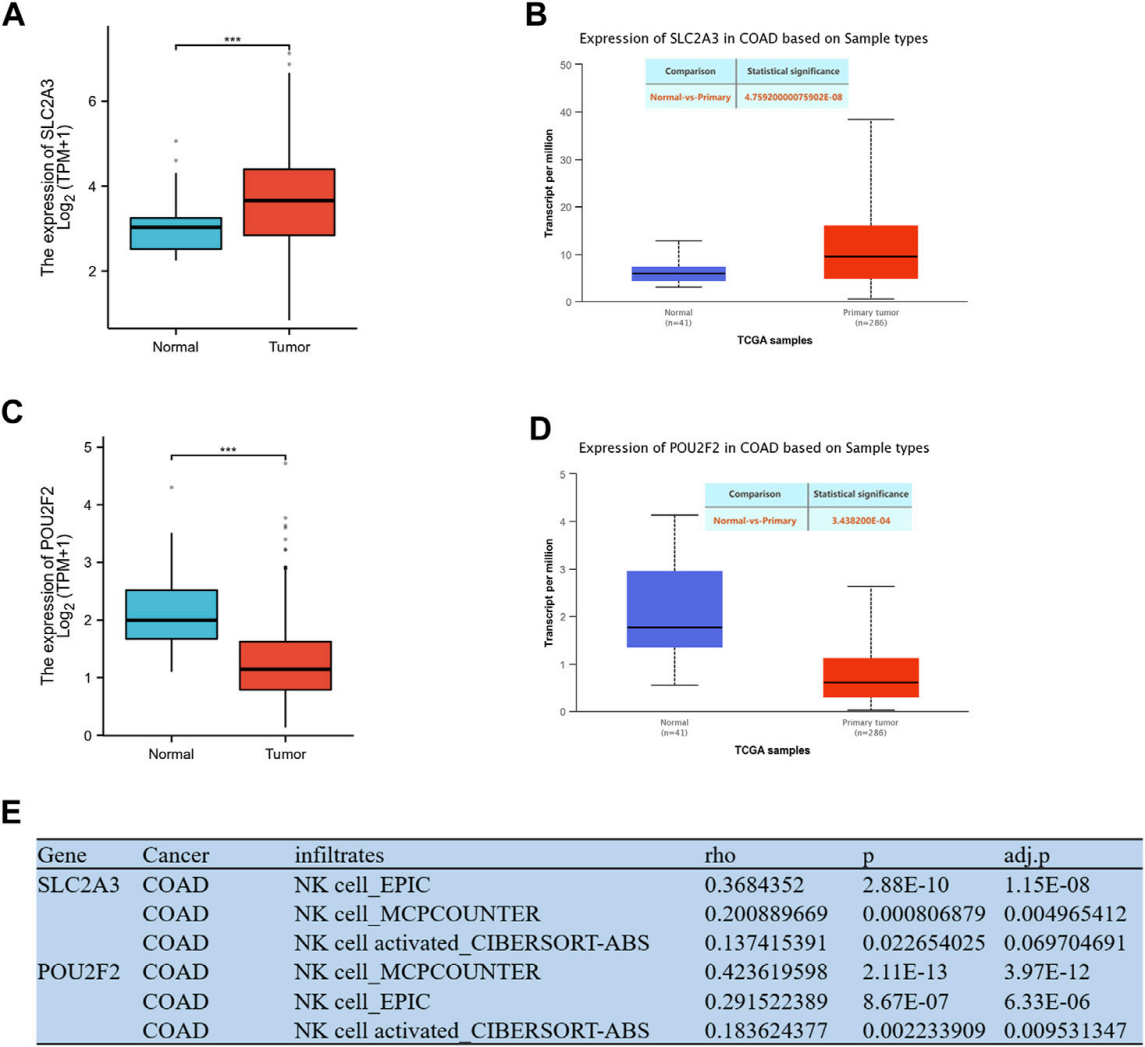
Expression levels of model genes and NK correlation. **(A)** The mRNA expression level of SLC2A3. **(B)** The protein expression level of SLC2A3. **(C)** The mRNA expression level of POU2F2. **(D)** The protein expression level of POU2F2. **(E)** Correlation analysis of genes and NK cell in the model. *** *p* < 0.001.

The gene expression levels were investigated using mRNA and protein expression profile data from TCGA-COAD. The results showed that the mRNA (Figure 5A) and protein (Figure 5B) expression levels of *SLC2A3* were significantly increased in tumor tissues, while the expression level of *POU2F2* (Figures 5C, D) was significantly decreased in tumor tissues. The TIMER web server was used to assess the association of *SLC2A3* and *POU2F2* with NK cells. Three different algorithms (EPIC, MCPCOUNTER and CIBERSORT-ABS) were used to estimate the correlation between genes and NK cells, and the results showed that these two genes both have a positive correlation with NK cells, and these two genes act as NKGs in CRC (Figure 5E).

### RS is an independent prognostic factor in CRC patients

**FIGURE 6:**
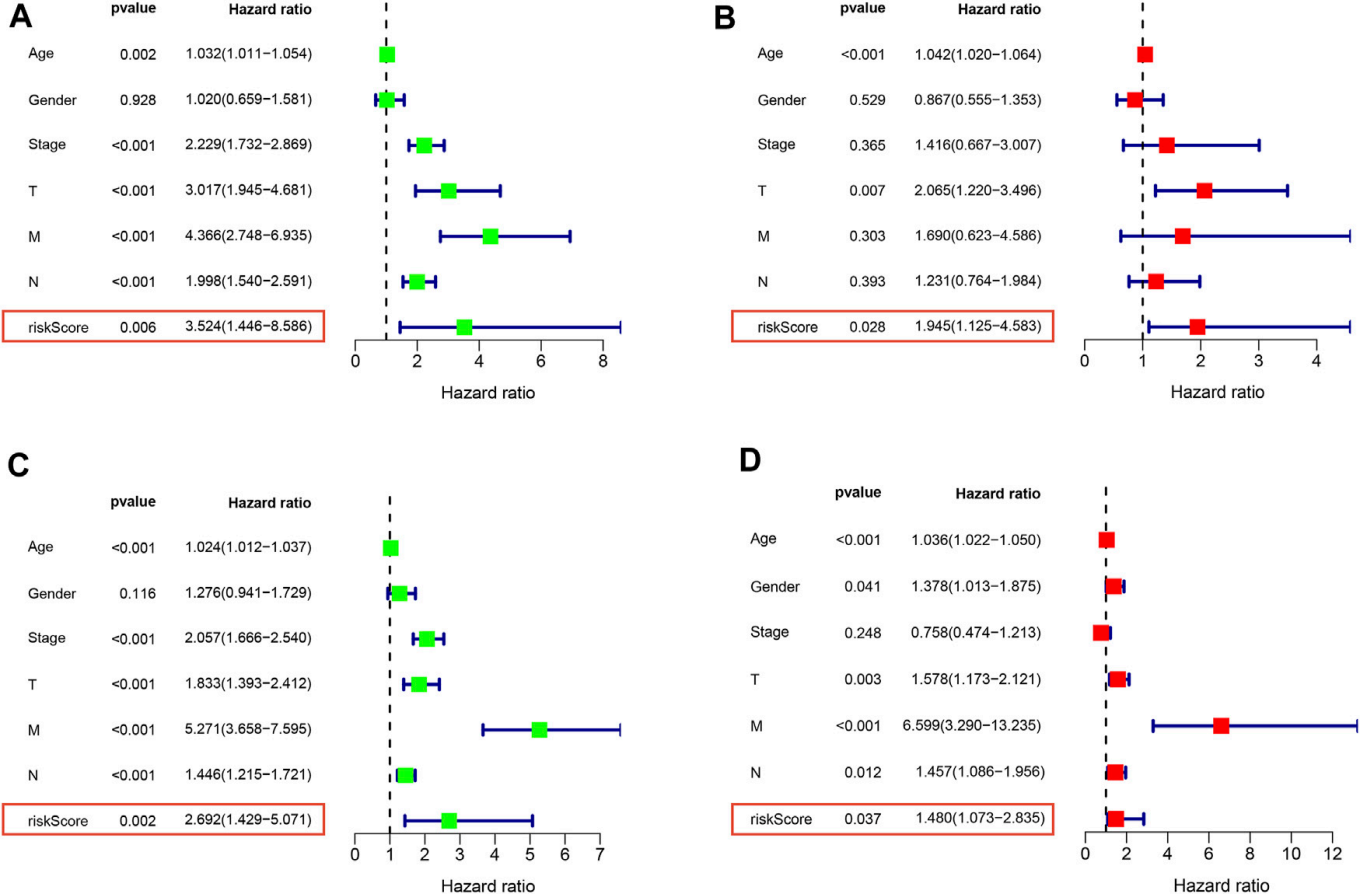
RS is an independent prognostic factor in CRC patients Univariate and multivariate COX analysis of TCGA **(A,B)** and GEO **(C,D)** patients. Left is univariate, and right is multivariate.

To further validate and develop the role of NKRS, we first tested whether RS is an independent prognostic factor in CRC patients by univariate and multifactorial COX analyses. The results showed that RS was its independent prognostic factor in both the TCGA-CRC cohort (Figures 6A, B) and the GSE39582 cohort (Figures 6C, D). GSEA enrichment analysis further revealed the enrichment pathway between high and low RS subgroups, results clarified that cell adhesion molecules (CAMs), chemokine signaling pathway, cytokine receptor interaction, focal adhesion, and systemic lupus erythematosus were mainly enriched in the high RS group (Supplementary Figures S3A), and ascorbate and adorate metabolism, butanoate metabolism, pentose, and glucuronate interconversions, peroxisome, and ribosome were mainly enriched in the low RS group (Supplementary Figures S3B). These findings inform further research and clinical application of RS.

### Validation based on hospital clinical patient data

**FIGURE 7:**
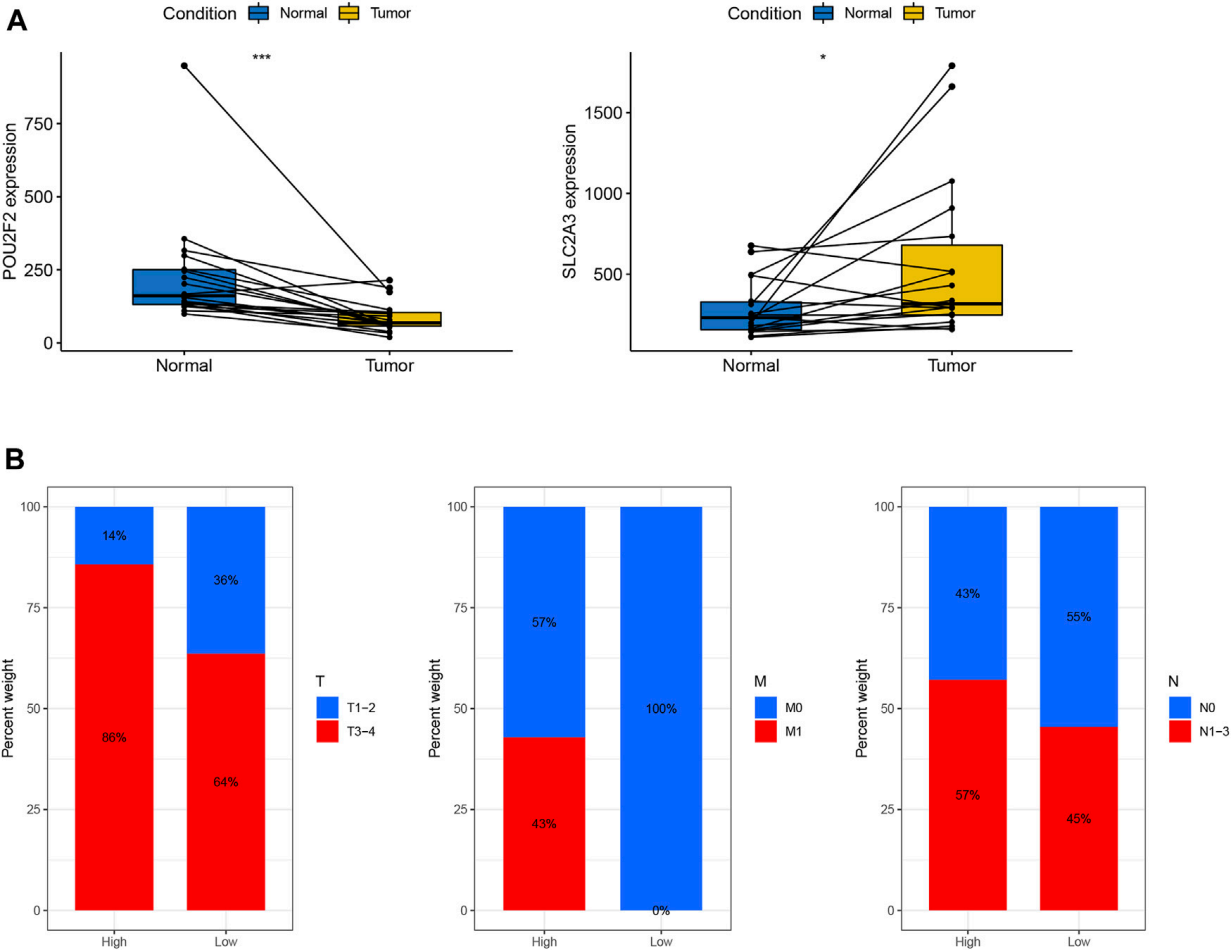
Validation based on hospital clinical patient data **(A)** Expression levels of POU2F2 and SLC2A3 in 18 paired colon cancer tissues. **(B)** Distribution of clinical patients (T, M, N) in high- and low-risk subgroups.

To further verify our results, we first collected fresh colon cancer tissues and normal tissues from 18 colon cancer patients without chemoradiotherapy from Guangxi Medical University Affiliated Cancer Hospital. Consistent with the results of the above analysis, which showed that the mRNA expression level of SLC2A3 in tumor tissues was significantly elevated, while the mRNA expression level of POU2F2 in tumor tissues was significantly reduced (Figure 7A). Furthermore, we differentiated patients into high and low RS subgroups according to their expression levels, and we found that more patients with advanced cancer were significantly enriched in the high RS group (Figure 7B). It is worth noting that no patients in the low RS subgroup developed metastasis, and all metastatic patients were enriched in the high RS subgroup. This may also be one of the reasons why patients with high RS have worse prognosis.

### Potential value of NKRS in the immunotherapy of CRC patients

**FIGURE 8:**
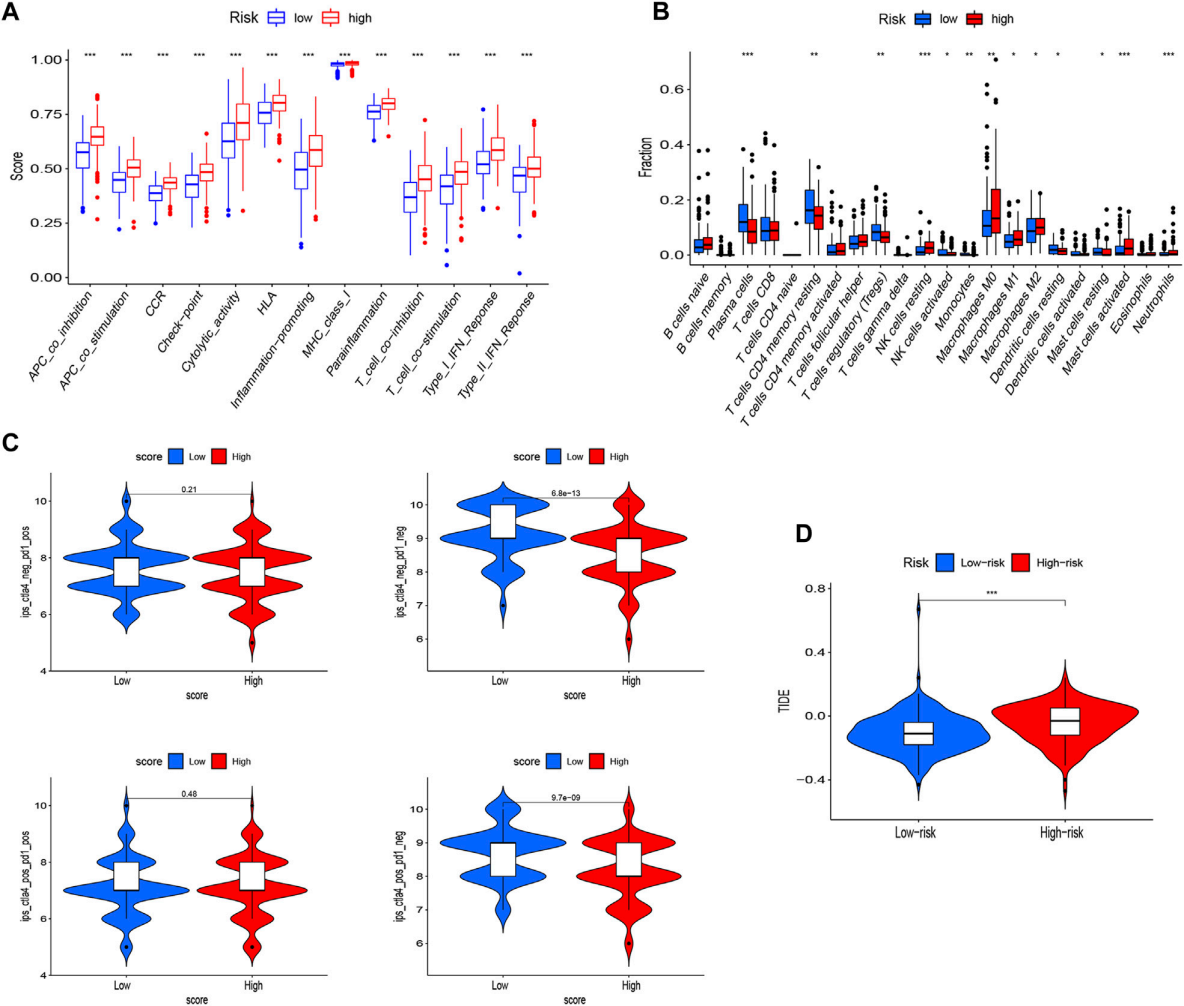
Potential application value of NKRS in immunotherapy Enrichment analysis of immune-related pathways **(A)** and immune cell infiltration **(B)** in high- and low-RS groups. **(C)** IPS scores for CTLA4− PD1+, CTLA4− PD1−, CTLA4+ PD1−, and CTLA4+ PD1+ in patients with high- and low-RS subgroups. **(D)** Prediction of immunotherapy response based on the TIDE algorithm. * *p* < 0.05, ** *p* < 0.01, and *** *p* < 0.001.

As a further investigation into the clinical relevance of NKRS, we looked at the proportion of higher- and lower-RS subgroups with the highest and lowest immune cell populations, respectively, by utilizing the ssGSEA algorithm. There was a significant enrichment of functional pathways associated with immunity in groups at high risk, such as Type-Ⅱ-IFN-Response, Type-Ⅰ-IFN-Response, T-cell-co-stimulation, T-cell-co-inhibition, Parainflammation, Inflammation-promoting, CCR, MHC-class-Ⅰ, APC-co-stimulation, Cytolytic-activity, HLA, Check-point, APC-co-inhibition (Figure 8A). On the other hand, the distribution of immune cells is more complicated, in which NK cells resting, Macrophages M2, Macrophages M1, Macrophages M0, and activated mast cells are significantly more common in the high-risk group, while plasma cells, T cells CD4 memory resting, T cells regulatory, and NK cells activated in low-risk individuals were significantly enriched (Figure 8B). IPS were used to predict patient responses to immunotherapy. Based on the study results, there is a higher likelihood of ctla4 sensitivity in the low RS group than in the high RS group (Figure 8C). The TIDE algorithm further validates our results. There was a significant difference in the TIDE score between low and high RS groups (Figure 8D), and this might suggest that low-RS groups respond better to immunotherapy. These results suggest that NKRS has one of the factors to identify patients’ responses to immunotherapy.

### NKRS has the potential to be a predictor of response to immunotherapy

**FIGURE 9:**
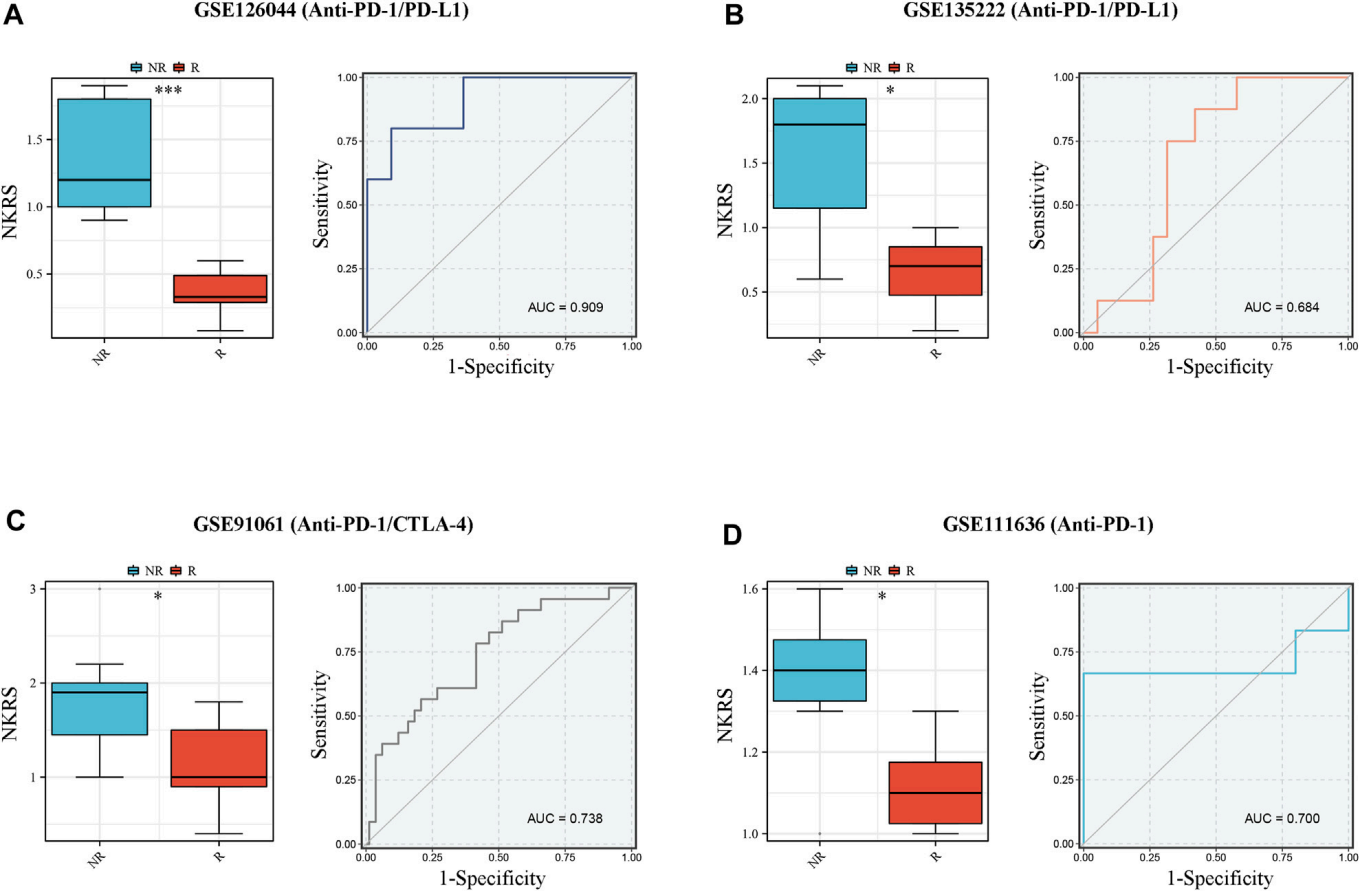
NKRS has the potential to be a predictor of response to immunotherapy. **(A–D)** R patients had lower risk score in all the four cohorts, and low risk group showed higher proportion of responders to anti-PD-1, anti-PD-L1, and anti-CTLA-4 immunotherapy. NR: not responding to immunotherapy. R: respond to immunotherapy. *p < 0.05 and ***p < 0.001.

In CRC patients we found that patients in the low NKRS group were more sensitive to immunotherapy. To further examine the role of NKRS in immunotherapy, we applied four external independent immunotherapy cohorts to assess the performance of NKRS in predicting immunotherapy response (Anti-PD-1/PD-L1/CTLA-4). The results showed that patients who responded to immunotherapy in all four different cohorts had a lower NKRS (Figures 9A–D). Furthermore, the results of AUC further demonstrated the accuracy of NKRS prediction. The results suggest that NKRS shows great potential in predicting immunotherapy treatment. Patients with low NKRS may be better suited for immunotherapy.

### SLC2A3 may be a potential therapeutic target in CRC patients

**FIGURE 10:**
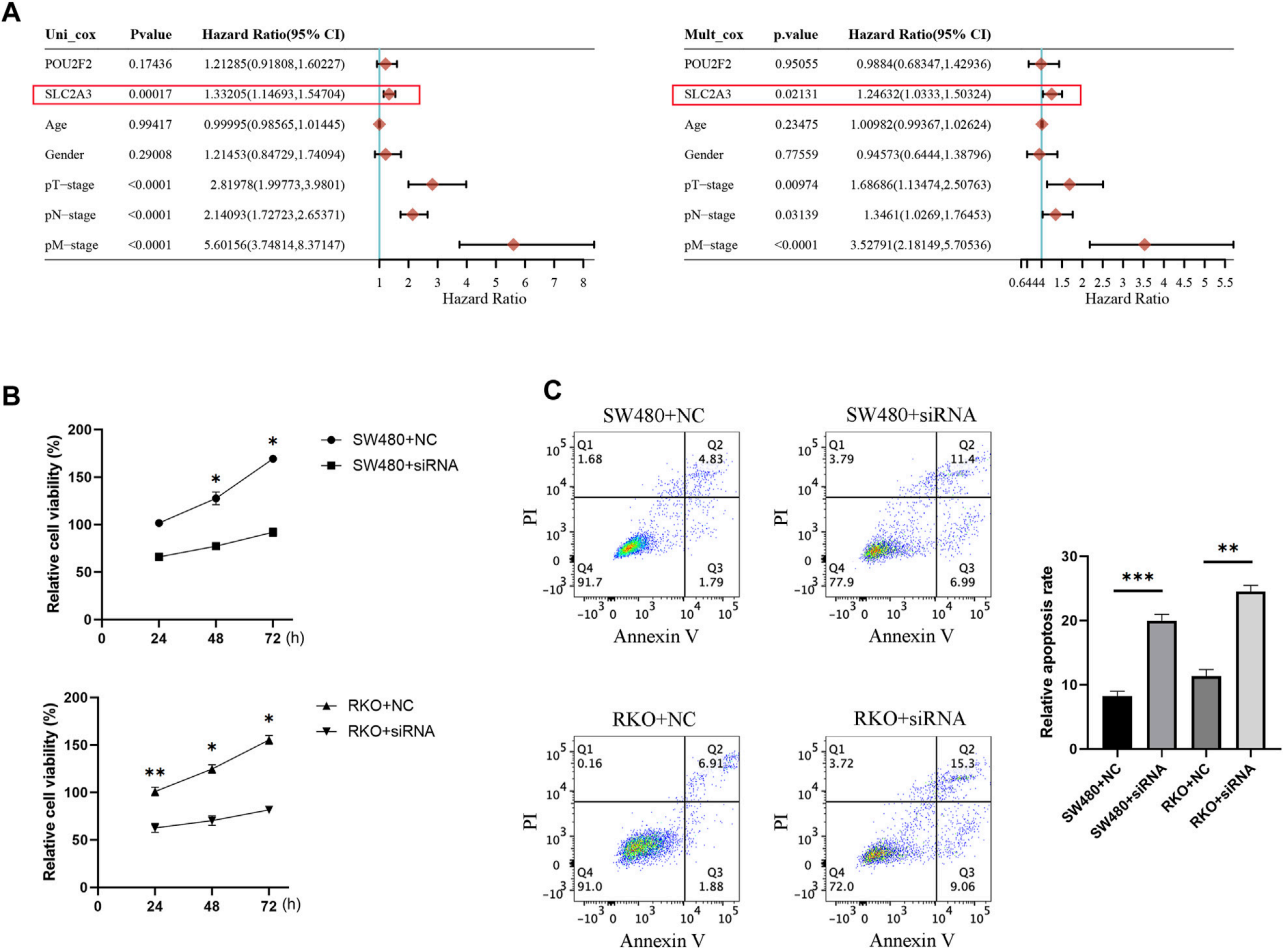
SLC2A3 may be a potential therapeutic target in CRC patients. **(A)** Univariate and multivariate COX analysis results indicated that SLC2A3 could serve as an independent prognostic factor in CRC patients. **(B)** Knockdown of SLC2A3 effectively inhibited the proliferation of CRC cells. **(C)** Knockdown of SLC2A3 promoted apoptosis of CRC cells. *N* = 3. * *p* < 0.05, ** *p* < 0.01, and *** *p* < 0.001.

**FIGURE 11:**
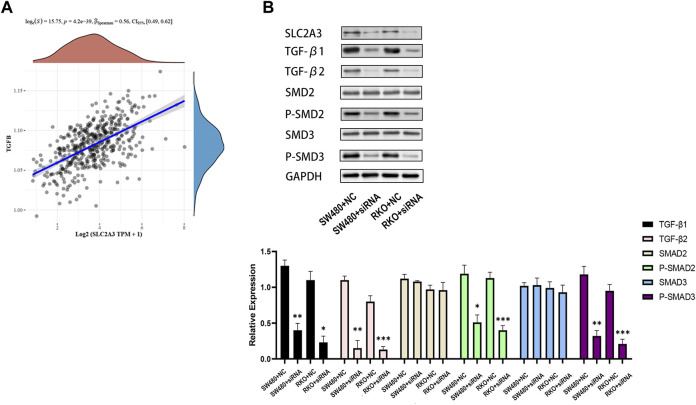
Dysregulated SLC2A3 is associated with the TGF-β signaling pathway. **(A)** SLC2A3 has a significant positive correlation with the TGF-β signaling pathway. **(B)** Knockdown of SLC2A3 can inhibit the activity of TGF-β signaling pathway in CRC cells. *n* = 3. * *p* < 0.05, ** *p* < 0.01, and *** *p* < 0.001.

Based on the published data of TCGA-CRC patients, the results of univariate and multivariate COX analysis indicated that SLC2A3 is an independent prognostic factor for CRC patients (Figure 10A). High expression of SLC2A3 may predict poor prognosis. In order to further study the potential biological function of SLC2A3 in CRC, we knocked down SLC2A3 by siRNA. The results showed that the proliferation rate of CRC cells was significantly decreased and the apoptosis of CRC cells was increased when SLC2A3 was knocked down (Figures 10B, C). In addition, the results of bioinformatics analysis showed that SLC2A3 was significantly related to TGF-β signaling pathway (Figure 11A). Aberrant activation of the TGF-β signaling pathway is often associated with a more malignant phenotype of the tumor. Western blot results showed that when SLC2A3 was knocked down, the expression levels of key proteins in the TGF-β signaling pathway were significantly inhibited (Figure 11B). Abnormally expressed SLC2A3 may maintain the malignant proliferation ability of CRC cells through TGFβ signaling pathway.

## Discussion

The NK cells are one of the most promising tools in cancer immunotherapy, as they can act against tumors without prior sensitization (Wu et al., 2020b). An NK cell can release cytotoxic granules containing the granzymes and cytotoxic enzymes perforin, leading to tumor cell lysis (Sconocchia et al., 2014; Krijgsman et al., 2019). NK cells also can kill cancer cells through CD16-mediated antibody-dependent cytotoxicity (ADCC). In addition, NK cells can activate adaptive immune responses by regulating interferon-gamma (IFN-γ) and tumor necrosis factor (TNF) (Tang et al., 2019; Wendel et al., 2021). Researchers have shown that NK cells are capable of treating a variety of cancers. Following the remarkable success of CAR-T cells in treating hematological malignancies, CAR-NK cells for cancer therapy have also gained attention in recent years (Wang et al., 2018; Sorrentino et al., 2021; Vuletić et al., 2021). Colon cancer is a common and highly malignant cancer. Its incidence increases rapidly, and the prognosis is poor. The development of immunotherapies may help in the treatment of CRC patients.

Two single-cell datasets of CRC were used in this study in order to identify genes related to NK regulation. The approach of modeling cancer through key genes has been widely used in cancer research (Wu et al., 2020c; Zhang et al., 2021b; Huang et al., 2023). Functional enrichment analysis revealed that these genes were mainly enriched in pathways related to immune regulation. Based on these NKGs, an NKGs prognostic model in CRC was developed and generated NKRS. We found that high NKRS was significantly associated with a poorer prognosis. Results from an external independent validation cohort and ROC curves further validate the accuracy of our model. NKRS was found to be the independent prognostic indicators in CRC patients by both univariate and multivariate COX analyses. In addition, we collected clinical tissues of 18 patients from Guangxi Medical University Affiliated Cancer Hospital. We validated the expression levels of genes in the NKRS model in patients. After dividing hospital patients into high and low RS subgroups, we found that patients with worse clinical features were significantly enriched in the high RS group. The prediction results of both IPS and TIDE algorithms suggest that the low-RS group of CRC patients is more sensitive to immunotherapy response. Several previous studies have also reported that NK-related characteristic models can predict immunotherapy response in patients, but few have focused on CRC patients and most have not been tested in external independent data (Cursons et al., 2019; Li et al., 2022a; Chi et al., 2022; Lan et al., 2023). We further collected four external independent immunotherapy cohorts (Anti-PD-1/PD-L1/CTLA-4) to assess the performance of NKRS in predicting immunotherapy response. The results showed that NKRS has good ability to predict patient immune response and that patients with low RS are more suitable for immunotherapy.

We further investigated the key genes that constitute the NKRS model and showed that SLC2A3 was significantly highly expressed in CRC, while the expression level of POU2F2 was significantly decreased. POU2F2 is thought to have a pro-oncogenic role in some cancers, but its role in CRC remains unclear (Katoh and Katoh, 2007; Yang et al., 2021). Kuo et al. showed that the upregulation of SLC2A3 expression level was associated with poorer prognosis in CRC patients (Kim et al., 2019). Our results based on unifactorial and multifactorial COX analysis suggest that SLC2A3 may be an independent prognostic factor in CRC. In order to further explore the potential biological function of SLC2A3, we knocked it down. The results showed that the knockdown of SLC2A3 in CRC cells significantly inhibited the proliferation and induced apoptosis of CRC cells. The study by Huabin et al. showed that SLC2A3 is highly enriched in the classical epithelial-mesenchymal transition (EMT) pathway and may be involved in PD-L1-mediated immune responses (Gao et al., 2021). GLUT3, the glucose transporter protein encoded by SLC2A3, was found to interact with Yes-associated protein (YAP) to promote CRC invasiveness and stemness (Kuo et al., 2019). In addition, the Glut3-YAP number pathway may also be associated with metabolic reprogramming in CRC (Kuo et al., 2019). GLUT3 is thought to be critical for resisting energy stress and enhancing the efficacy of current colorectal cancer therapies (Dai et al., 2020).

The results of bioinformatics analysis indicated that SLC2A3 had a potential correlation with the TGF-β signaling pathway. Molecular pathways of TGF-β signaling are typical pathways that regulate tumorigenesis and tissue homeostasis and have been extensively studied in various biological processes. It has dual roles as a tumor suppressor in precancerous cells and a tumor promoter in cancer cells and has been shown to be involved in the development of various malignancies including CRC (Gachpazan et al., 2019; Tang et al., 2020). The results of Western blot showed that the expression and phosphorylation levels of key proteins in the TGF-β signaling pathway were significantly affected when SLC2A3 was knocked down. SLC2A3 may participate in CRC process through TGF-β signaling pathway. TGF-β signaling in colon cancer cells can promote immunosuppression within the tumor microenvironment, which can inhibit the activation and function of immune cells such as T cells and natural killer cells, thereby evading immune surveillance and promoting tumor immune escape (Rizzo et al., 2011; Li et al., 2022b). Our results suggest that SLC2A3 knockdown can inhibit the activity of the TGF-β signaling pathway, and thus SLC2A3 may be a new target for CRC therapy.

## Conclusion

This study developed and validated a prognostic model of NKGs in CRC patients based on single-cell dataset of CRC patients. The model-generated NKRS may be useful for personalizing therapy and predicting the immune response of patients. Furthermore, the key gene SLC2A3 in the model has the potential to be a biomarker for CRC patients.

## Data Availability

The original contributions presented in the study are included in the article/Supplementary Material, further inquiries can be directed to the corresponding authors.

## Supplementary material

SUPPLEMENTARY FIGURE S1 Model establishment. **(A)** Identification of genes that are significantly different in CRC patients. **(B)** Heatmap of DEGs. **(C)** Cross‐validation for tuning parameter selection in the lasso regression. **(D)** Validation was performed for tuning parameter selection through the least absolute shrinkage and selection operator (LASSO) regression model for overall survival (OS).

SUPPLEMENTARY FIGURE S2 Analysis results of PCA.

SUPPLEMENTARY FIGURE S3 GSEA enrichment analysis results in high **(A)** and low **(B)** RS groups.

## Abbreviations

CAR, chimeric antigen receptors; CRC, colorectal cancer; DEGs, differentially expressed genes; GEO, Gene-Expression Omnibus; NK, Natural killer; NKRS, NK-related risk scores; OS, Overall survival; ROC, receiver operating characteristic; TCGA, The Cancer Genome Atlas; TME, Tumor microenvironment.
