# Supplementary material for: Natural killer cell-related prognosis signature predicts immune response in colon cancer patients
Source: Front Pharmacol. 2023 Nov 13;14:1253169. doi: 10.3389/fphar.2023.1253169 (PMC10679416; doi:10.3389/fphar.2023.1253169)
Supplement: Supplementary file 1 [file Table1.DOCX]

**Supplementary Table 1.** NK-related genes in CRC were identified based on two single-cell data

| Gens |
| --- |
| ACTN4 |
| ARPC2 |
| FASLG |
| FGL2 |
| MATK |
| GZMM |
| SOD2 |
| MS4A1 |
| CD4 |
| RNASE6 |
| PSAP |
| PTGER2 |
| HERPUD1 |
| RUNX3 |
| GPR183 |
| CD160 |
| CLIC3 |
| ATF3 |
| RHOC |
| GZMA |
| SPI1 |
| CST7 |
| NPC2 |
| CD79A |
| NR4A1 |
| LDLRAD4 |
| DBI |
| CFL1 |
| SLC2A3 |
| SAMSN1 |
| FCN1 |
| IFI30 |
| GLUL |
| CD74 |
| CTSW |
| ANKRD28 |
| CDK2AP2 |
| TNFRSF1B |
| LY86 |
| GZMB |
| CYBB |
| PRDX4 |
| HSPE1 |
| CST3 |
| NEAT1 |
| CD247 |
| GRN |
| TRG-AS1 |
| SERPINA1 |
| CHST12 |
| ID2 |
| TCF4 |
| HLA-DQB1 |
| EGR1 |
| SH2D1B |
| ITM2A |
| GNG7 |
| 1-Mar |
| MS4A6A |
| SH2D2A |
| NKG7 |
| HSPH1 |
| IFITM2 |
| KLRC1 |
| MS4A7 |
| SYNGR2 |
| CCL5 |
| FTH1 |
| CTSB |
| CTSS |
| ENTPD1 |
| HLA-DMA |
| KLRK1 |
| HLA-DRB1 |
| MARCKSL1 |
| IL32 |
| SGK1 |
| PKM |
| HSPA1B |
| LTB |
| LY6E |
| LYZ |
| HLA-DMB |
| CD7 |
| LDHA |
| CD27 |
| DOK2 |
| TNFRSF17 |
| MPEG1 |
| CAPG |
| STARD3NL |
| PLEKHF1 |
| NCF1 |
| PFN1 |
| UBE2J1 |
| NDUFB2 |
| CD83 |
| CCL4 |
| PRF1 |
| CTSH |
| CD79B |
| SRGN |
| ITGB2 |
| GAPDH |
| CD3D |
| GPX1 |
| AREG |
| IFITM1 |
| S100A8 |
| PLAUR |
| AIF1 |
| SPINT2 |
| SYTL3 |
| IL2RB |
| POU2F2 |
| FKBP1A |
| ABI3 |
| KLRD1 |
| TMEM176B |
| SAT1 |
| CD68 |
| CCR7 |
| XCL2 |
| RGS1 |
| PRDM1 |
| KLRB1 |
| S100A9 |
| MEF2C |
| HOPX |
| BIRC3 |
| PGK1 |
| GNLY |
| DERL3 |
| CD14 |
