# Supplementary figures and images for: Natural killer cell-related prognosis signature predicts immune response in colon cancer patients
Source: Front Pharmacol. 2023 Nov 13;14:1253169. doi: 10.3389/fphar.2023.1253169 (PMC10679416; doi:10.3389/fphar.2023.1253169)

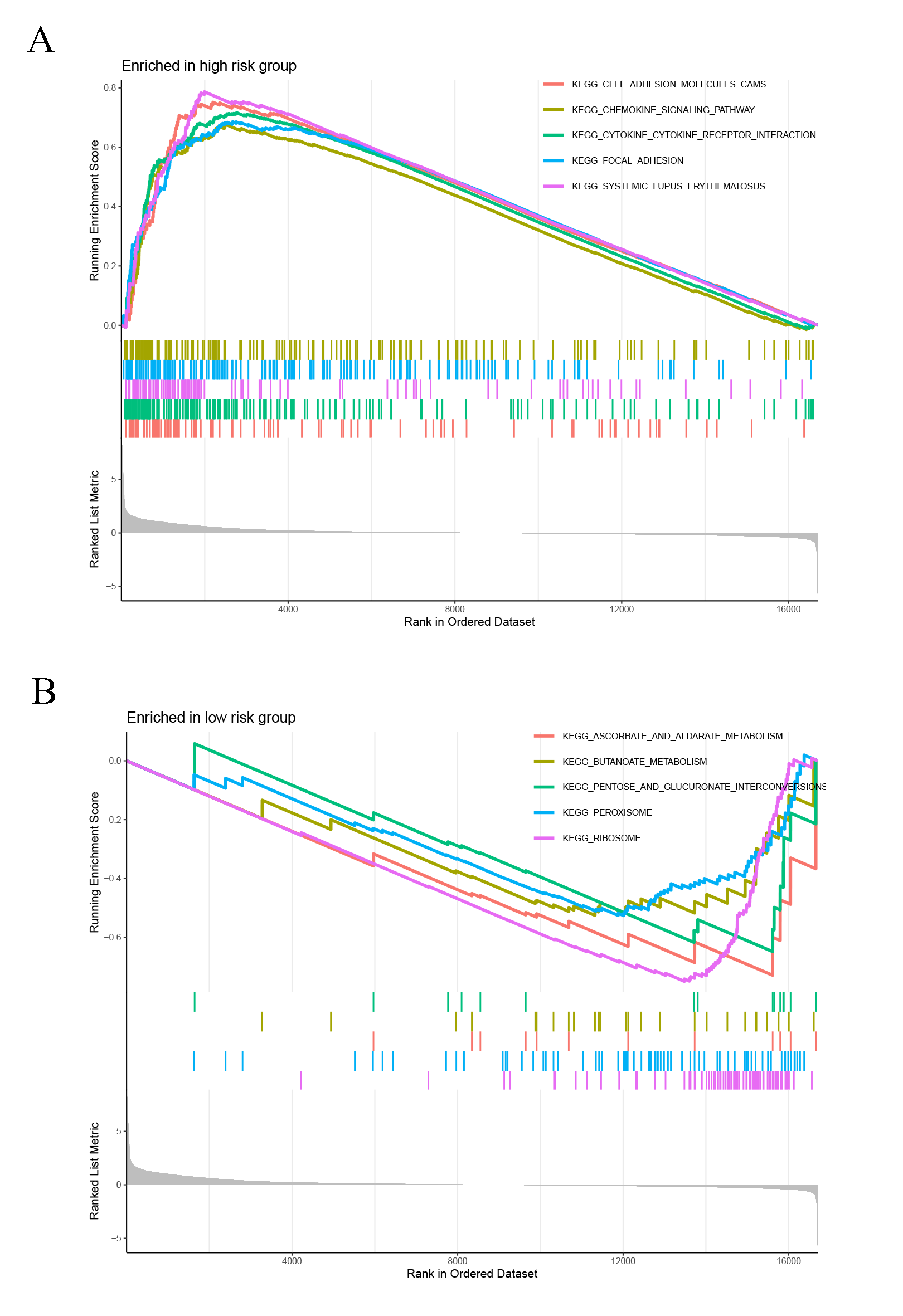

Supplement: Supplementary file 2 [file Image3.TIF]

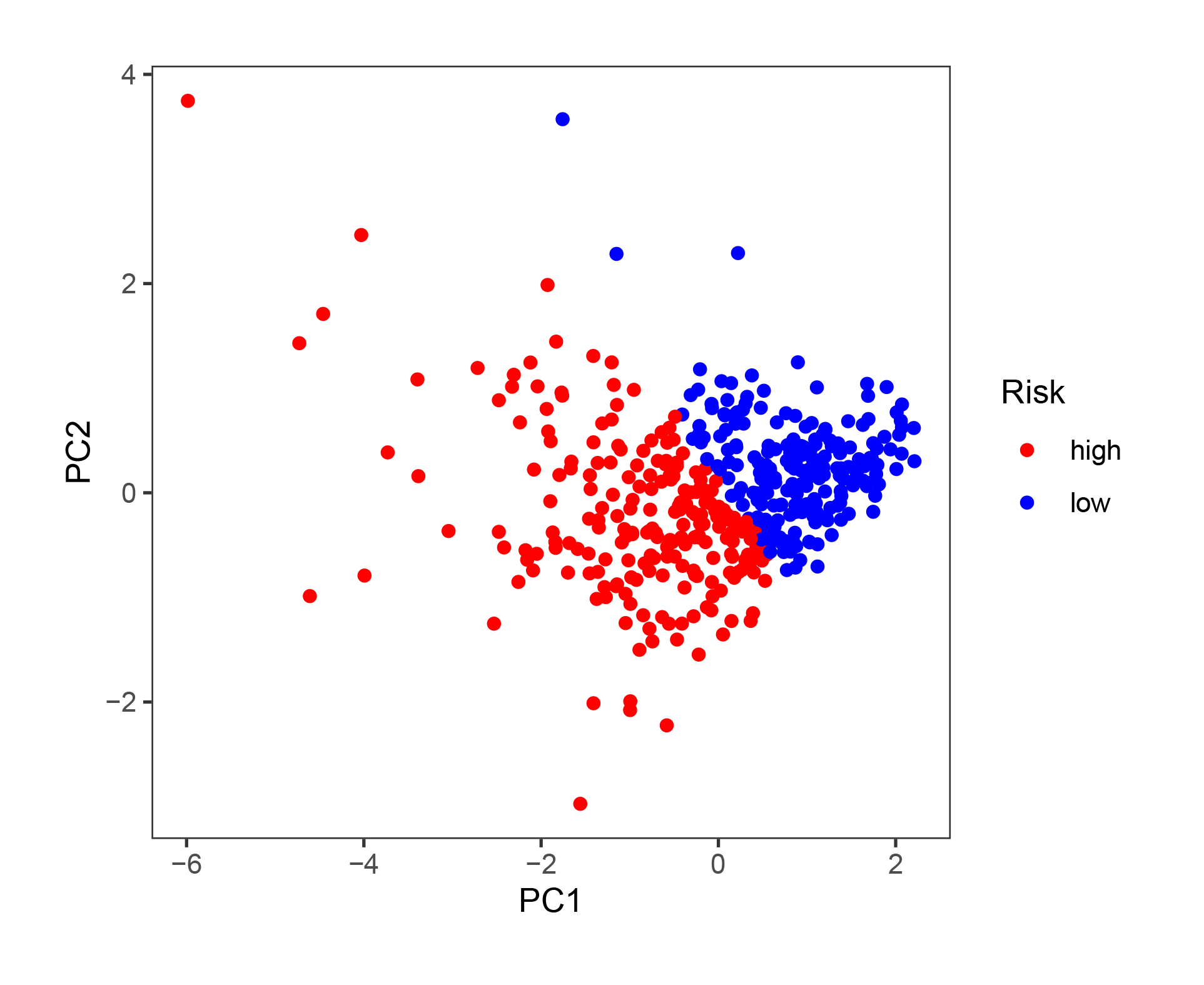

Supplement: Supplementary file 3 [file Image2.TIF]

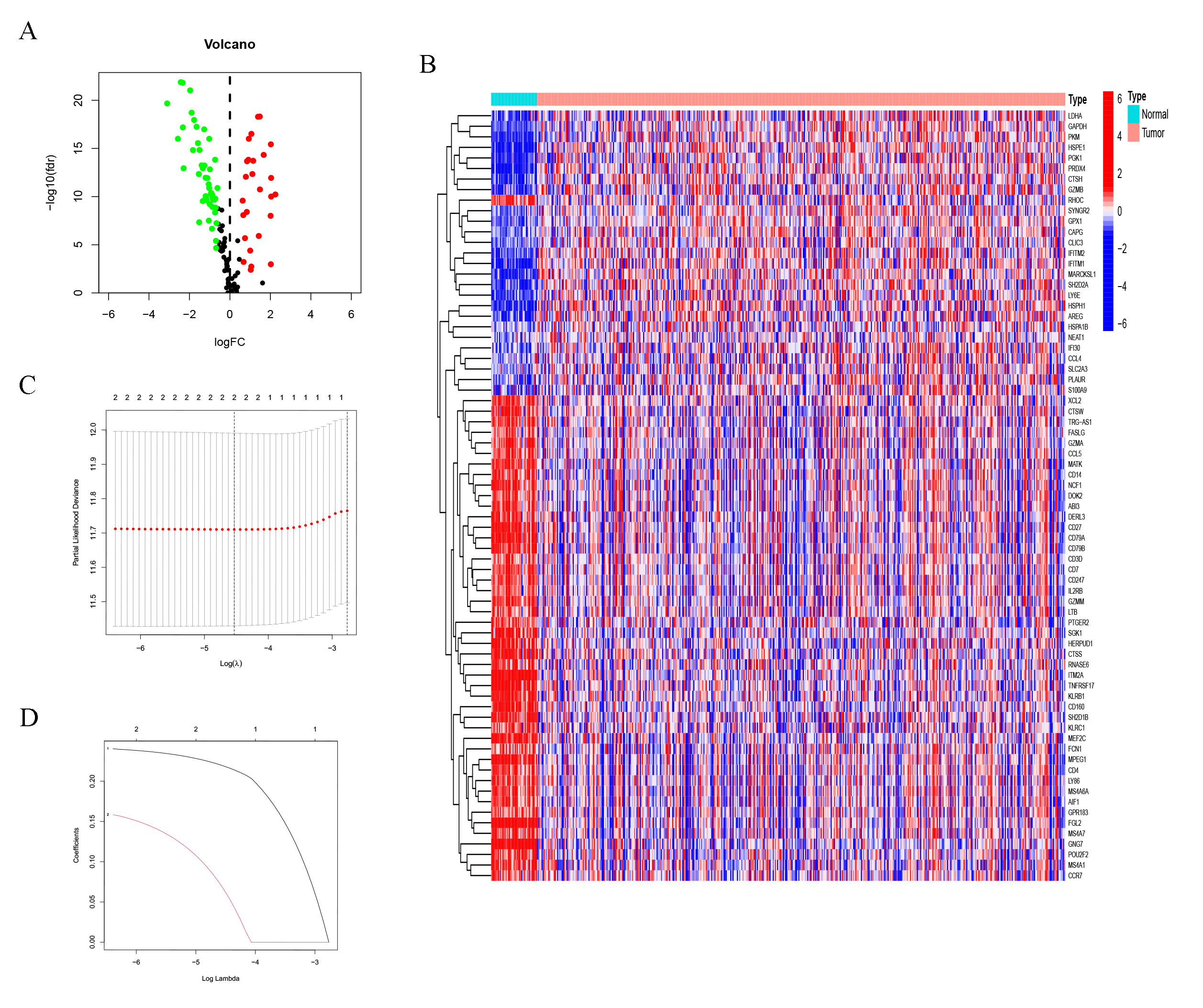

Supplement: Supplementary file 4 [file Image1.TIF]
